# Intravenous Immunoglobulin Reduces Infections for Patients with Chronic Lymphocytic Leukemia: A Single-Center Retrospective Analysis

**DOI:** 10.1158/2767-9764.CRC-26-0177

**Published:** 2026-05-18

**Authors:** Nirja N. Shah, Tulsi Patel, Thomas J. Kipps, Michael Y. Choi

**Affiliations:** 1 Department of Medicine, UC San Diego, La Jolla, California.; 2 Division of Hematology and Cellular Therapy, Department of Medicine, UC San Diego, La Jolla, California.; 3 Center for Novel Therapeutics, La Jolla, California.; 4 UC San Diego Sanford Stem Cell Institute, La Jolla, California.

## Abstract

Hypogammaglobulinemia is common in patients with chronic lymphocytic leukemia (CLL). Prior studies demonstrated that intravenous immunoglobulin (IVIG) reduced infections without survival benefit. We conducted a single-institution retrospective cohort study of infectious outcomes in patients with CLL who received IVIG between 2005 and 2022 to assess the impact of IVIG across evolving CLL treatment paradigms, including the introduction of targeted agents. Fifty-two patients met the inclusion criteria of IVIG use for hypogammaglobulinemia and recurrent infections, with also any infection up to 1 year prior to the initiation of IVIG. Baseline mean IgG prior to IVIG was 389 mg/dL (range, 93–891 mg/dL). By design, all 52 patients (100%) experienced at least one CTCAE grade ≥2 infection in the 12 months prior to IVIG initiation. In the 12 months following IVIG initiation, only 17 patients (33%) experienced a grade ≥2 infection. IVIG therapy was associated with a 67% reduction in risk of grade 2 infections (RR 0.33; 95% confidence interval, 0.22–0.48; *P* < 0.0001), corresponding to an absolute risk reduction of 67% and a number needed to treat of 1.5. The mean number of infections per patient also reduced from 2.1 to 0.4. Baseline IgA and IgG levels, as well as recent CLL treatment, were not predictors of residual post-IVIG infections. These findings suggest that IVIG may yield meaningful improvements in patient quality of life by reducing infections.

**Significance:**

Hypogammaglobulinemia and recurrent infections remain a complication of CLL, despite contemporary agents. In this 2005 to 2022 retrospective cohort, IVIG initiation was associated with a large reduction in CTCAE grade ≥2 infections: only 33% of patients had a post-IVIG grade ≥2 infection versus all patients before IVIG. Residual infections were not explained by baseline IgG/IgA levels or recent therapy. IVIG may be an effective supportive-care strategy in patients with CLL.

## Introduction

For patients with chronic lymphocytic leukemia (CLL), infections remain a major cause of morbidity and mortality (1). Mechanisms of immunocompromise include both cellular and humoral immune deficiencies, as well as potential effects of therapy. Hypogammaglobulinemia is present in 20% to 60% of patients at diagnosis, often worsens over the disease course, and is associated with an increased risk of infectious complications (2, 3).

The National Comprehensive Cancer Network (NCCN) recommends IgG replacement therapy (IgRT) for patients with CLL with recurrent, severe sinopulmonary infections requiring intravenous antibiotics or hospitalization in the setting of hypogammaglobulinemia (e.g., serum IgG <500 mg/dL). It is recommended that intravenous immunoglobulin (IVIG) is typically dosed monthly at 0.3 to 0.5 g/kg body weight, with adjustment of dose and interval to maintain a nadir IgG level of approximately 500 mg/dL (4).

The evidence supporting the use of IgRT in patients with CLL is largely derived from studies in which patients had been treated with chemotherapy agents like fludarabine or alkylating agents. A 1988 trial demonstrated reduced bacterial infections with IVIG in patients with CLL with hypogammaglobulinemia (IgG ≤50% of the lower limit of normal; ref. 5). A subsequent trial in 1995 confirmed these findings (6). A 2008 Cochrane meta-analysis of these and other randomized trials in lymphoproliferative disorders concluded that although IgRT did not confer a survival benefit, it significantly reduced documented infections in patients with hypogammaglobulinemia and recurrent infections (7).

However, the impact of IgRT on infection reduction has not been studied extensively in the context of newer targeted therapies. The immunologic consequences of Bruton’s tyrosine kinase inhibitors, venetoclax, or other CD20 monoclonal antibody combination are not as well established. For example, ibrutinib-treated patients with CLL showed improvements in IgA that correlated with a reduced rate of infections, whereas IgG declined only after longer-term treatment (8). Although contemporary targeted therapies may result in less immune suppression than prior purine analog therapies, infections remain a problem for patients with CLL. These findings highlight the need to reassess the role of IgRT in the context of targeted treatments.

## Materials and Methods

This retrospective cohort study included patients with CLL treated at a single academic institution between 2005 and 2022. The study was approved by the University of California San Diego Institutional Review Board under protocols #171884 and #080918. This study was reviewed and approved in accordance with the US Common Rule; informed consent was waived because of the retrospective nature of the study. Patients had previously provided written informed consent for CLL-related research. Patients were identified based on documented receipt of IgRT and excluded if IgRT was administered for indications other than infection prophylaxis or if documentation was insufficient to assess infectious outcomes.

Each patient was systematically reviewed through the electronic medical record for demographic characteristics, CLL disease characteristics, baseline IgG and IgA levels, and concurrent CLL-directed therapies at the time of IVIG administration. IVIG initiation criteria were not standardized; decisions were made at the treating physician’s discretion, most commonly prompted by recurrent infections and low serum IgG levels. Once initiated, all patients received IVIG at 0.4 g/kg every 28 days per NCCN guidelines. A subset of patients initiated IgRT despite IgG levels above commonly cited thresholds, reflecting clinician concern for overall immune dysfunction.

Infectious outcomes were evaluated during two predefined 12-month observation periods: before and after IgRT initiation. The infection severity was classified per the Common Terminology Criteria for Adverse Events (CTCAE) version 5.0. Grade 2 infections were defined as infections requiring oral antimicrobial therapy or outpatient management, whereas grade 3 infections were defined as infections requiring hospitalization or intravenous antimicrobials. Multiple clinical encounters related to the same infectious episode were counted as a single event.

For statistical analysis, baseline characteristics were summarized using descriptive statistics. The primary endpoint was occurrence of at least one CTCAE grade ≥2 infection within each window. Risk ratios with 95% confidence intervals (CI) were calculated; absolute risk reduction and number needed to treat were derived. Exploratory analyses comparing patients with and without post-IgRT infections used *χ*^2^/Fisher exact tests for categorical variables and two-sample *t* tests/Wilcoxon rank-sum tests for continuous variables. The time to the first post-IgRT infection was analyzed using a swimmer plot and the Kaplan–Meier method, with patients censored at 365 days. Analyses were performed using standard statistical software.

## Results

Of 137 patients with CLL who received IgRT, 65 met inclusion criteria (IgRT administration for hypogammaglobulinemia or recurrent/clinically significant infections) and had sufficient chart data available for detailed review. The final analytic cohort comprised 52 patients who had experienced at least one grade ≥2 infection in the year preceding IVIG initiation; the remaining 13 were started on IVIG primarily for history of infections that predated the 1-year pre-IVIG window. All patients received IVIG at 0.4 g/kg every 28 days per NCCN guidelines. No patients required dose adjustments during the 12-month observation period.

The median age at IVIG initiation was 68 years, with a mean interval from CLL diagnosis to IVIG initiation of 9.3 years. At IVIG initiation, 60% had early-stage disease (Rai stages 0–II) and 40% had advanced disease (Rai stages III–IV). Mean baseline IgG, drawn within 1 week prior to IVIG initiation, was 389 mg/dL (range, 93–891 mg/dL), and mean IgA was 42 mg/dL (range, 4–177 mg/dL). Of the 52 patients, 39 (75%) had baseline IgG below 500 mg/dL, consistent with the threshold commonly used to guide IVIG initiation. Of the remaining 13 patients started on IVIG despite IgG ≥500 mg/dL, most had levels <650 mg/dL and were treated due to concern for recurrent infections and immune dysfunction. Seventy-seven percent had received prior or concurrent CLL therapy at the time of IVIG initiation (Table 1).

**Table 1.** Baseline patient characteristics.

Characteristic	Value (*n* = 52)
Median age at CLL diagnosis, years	58 (IQR 52–64)
Median year of CLL diagnosis	2004 (IQR 2002–2009)
Median year of IVIG start	2014 (IQR 2010–2018)
Sex	​
Female	23 (44%)
Male	29 (56%)
Race	​
White, non-Hispanic	51 (98%)
Other/unknown	1 (2%)
Rai stage at IVIG start	​
0	0 (0%)
I	4 (8%)
II	27 (52%)
III	8 (15%)
IV	13 (25%)
Treatment within 1 year prior to or during IVIG start	​
CD20 mAb/CD20 combination^a^	9
BTK inhibitor	6
Alemtuzumab	3
Venetoclax	1
CD19 CAR-T cell	1
Inebilizumab (CD19 mAb, MEDI-551)	1

Abbreviations: BTK, Bruton’s tyrosine kinase; CAR, chimeric antigen receptor; IQR, interquartile range; mAb, monoclonal antibody.

a Includes three patients treated with rituximab + lenalidomide; two rituximab + methylprednisolone; two obinutuzumab + methylprednisolone; one obinutuzumab + chlorambucil; one fludarabine + cyclophosphamide + rituximab; and one rituximab monotherapy.

### Infection burden prior to IVIG initiation

In the 12 months before IVIG, all patients experienced at least one grade ≥2 infection (mean 2.1, range 0–4), predominantly upper respiratory infections with few urinary tract infections.

Although only four patients had a grade 3 infection within the pre-IVIG year, eight others had prior history of at least one grade 3 infection since being diagnosed with CLL. In total, 12 patients (23%) had experienced at least one grade 3 infection at any point since CLL diagnosis. No grade 4 or 5 infections occurred before IVIG.

### Infection outcomes following IVIG initiation

Following IVIG initiation, there was a marked reduction in infectious events. After IVIG, only 17 patients (33%) experienced a grade ≥2 infection (mean 0.4 per patient). The grade 2 infections consisted mostly of sinusitis or upper respiratory infections. Two patients (4%) experienced grade 3 infections (one multifocal pneumonia and one cellulitis); no grade 4 or 5 events occurred.

Compared with the pre-IVIG period, IVIG therapy was associated with a 67% relative risk reduction in grade ≥2 infections (RR, 0.33; 95% CI, 0.22–0.48; *P* < 0.0001), an absolute risk reduction of 67%, and a number needed to treat of 1.5. Within the 12-month pre-IVIG window, 4 of 52 (8%) patients experienced a grade 3 infection, compared with 2 of 52 (4%) in the 12 months following IVIG initiation.

Twenty-one patients had received any CLL therapy prior to IVIG initiation, and seven patients received new CLL therapy specifically within 1 year of starting IVIG. No statistically significant differences were observed between patients with and without post-IVIG infections regarding prior CLL therapy (*P* = 0.91), baseline IgG (*P* = 0.79), or IgA (*P* = 0.22).

On the swimmer plot, 17 of 52 patients (33%) experienced at least one grade ≥2 infection over the 365-day post-IVIG follow-up. Of these, 15 patients (29%) experienced grade 2 infections only, with the time to first event ranging from 25 to 356 days (median 150 days). Two patients (4%) developed grade 3 infections at days 43 and 208, respectively; neither had a concurrent grade 2 infection during follow-up. The remaining 35 patients (67%) completed follow-up without any grade 2 or 3 infection. Events were distributed across the full observation window, though a notable proportion occurred within the first 120 days.

**Figure 1.**
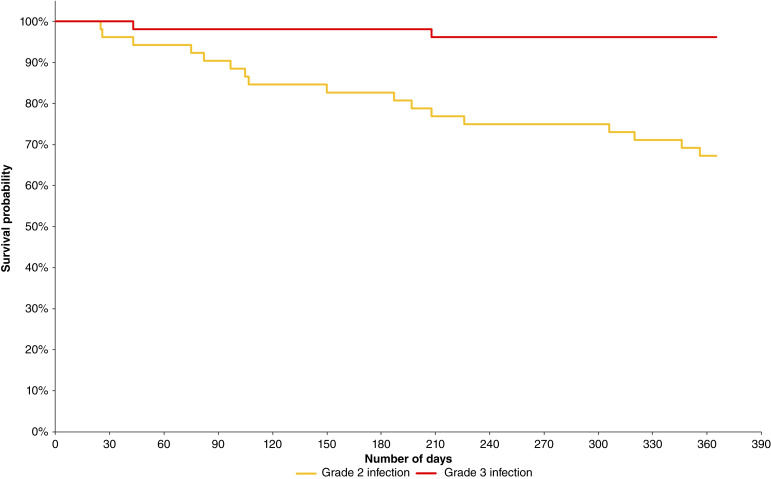
Kaplan–Meier analysis. Kaplan–Meier analysis demonstrates a gradual, relatively uniform decline in grade 2 infection-free survival over 365 days.

Kaplan–Meier analysis (Fig. 1) demonstrated a gradual, relatively uniform decline in infection-free survival over 365 days, suggesting a constant hazard of infection rather than a period of heightened vulnerability. The estimated 1-year infection-free survival probability was 67.3% for any infection (grade 2 or 3 combined). In contrast, the grade 3 infection-free survival curve remained nearly flat at 96.2%, reflecting the rarity of severe infections.

Among the 39 patients with baseline IgG <500 mg/dL, all 39 (100%) experienced at least one grade 2 or higher infection in the year prior to IVIG initiation. Specifically, 38 (97%) had at least one grade 2 infection, and three (8%) had at least one grade 3 infection; of the latter, two patients experienced both grade 2 and grade 3 infections, whereas one experienced a grade 3 infection without a concurrent grade 2 infection. Following IVIG initiation, 11 patients (28%) experienced a grade 2 infection and no patients experienced a grade 3 infection, representing a significant reduction in infection burden in this subgroup.

In the overall cohort of 52 patients, trough IgG levels were examined in relation to post-IVIG infection outcomes. Among the 17 patients who developed infections after IVIG initiation, the median preinfusion IgG trough nearest to the infectious event was 703 mg/dL; 6 had trough IgG <500 mg/dL, and 11 had trough IgG ≥500 mg/dL. Among the 35 patients who remained infection-free, the median IgG nadir during follow-up was 687 mg/dL, with 30/35 maintaining levels ≥500 mg/dL.

## Discussion

In this intrapatient pretreatment/posttreatment analysis, IVIG prophylaxis was associated with a substantial reduction in grade 2 infections, from 100% before IVIG to 33% after IVIG. Grade 3 infections were too infrequent to draw strong conclusions.

The near-universal infection burden among patients with baseline IgG <500 mg/dL prior to IVIG initiation and the dramatic reduction in infections following initiation support the use of this threshold as a clinically meaningful trigger for prophylactic IVIG in patients with CLL, consistent with existing guidelines. However, patients with frequent infections and IgG levels only slightly above this arbitrary threshold may still benefit from IVIG.

The swimmer plot demonstrated that infections were distributed broadly across follow-up rather than clustering within a discrete window, and the majority of patients (67%) completed the observation period infection-free. Kaplan–Meier analysis showed the median time to first infection was not reached, supporting a favorable infection profile under IVIG. However, the steady, nonplateauing decline in infection-free survival suggests that IVIG does not fully eliminate infection susceptibility, and ongoing surveillance is warranted. Correcting hypogammaglobulinemia addresses only one aspect of the immune dysfunction in CLL.

These findings are directionally consistent with prior literature supporting IgRT in patients with CLL with hypogammaglobulinemia and recurrent infections prior to the era of targeted CLL therapies. A recent Australian cohort study (*n* = 6,217) found no reduction in serious infection-related hospitalizations among regular IgRT users. However, serious infections clustered around periods of IgRT initiation/reinitiation and discontinuation, suggesting that intermittent use may confound interpretation of efficacy (9). Furthermore, infection occurrence was significantly associated with survival in that cohort, suggesting that IVIG’s potential impact on survival should not be dismissed. In contrast, our study benefited from detailed review of individual medical records, allowing for more granular assessment of lower-grade infections, which remain clinically meaningful for patients with CLL. Although all patients in our cohort remained on IVIG, which is potentially necessary to fully achieve infection risk reduction, our findings suggest that continuous IgRT can reduce moderate infections. Our results highlight the value of patient-level evaluation.

Limitations include the single-center retrospective design with possible incomplete capture of infections treated externally. Our study period (2005–2022, median IVIG start 2014) means a substantial portion of patients initiated IVIG before widespread adoption of targeted therapies such as ibrutinib and venetoclax. Although this limits our ability to draw conclusions specific to the targeted therapy era, the inclusion of patients across treatment paradigms enhances generalizability to the broader CLL population. We could not standardize IVIG dosing and adherence or trough response and therefore cannot determine whether outcomes varied by frequency of IVIG infusions or total number of IVIG infusions received in 1 year. Furthermore, the COVID-19 pandemic may have influenced temporal infection patterns. Lastly, as the patients in our cohort continued IVIG on a monthly basis, we did not evaluate the effects of IVIG discontinuation. Whereas some treatment guidelines support temporary cession of IVIG based on trough levels (4), all of our patients continued IVIG for the entire duration of follow-up.

We and others have found that the primary impact of IVIG is reduction of grade 2 infections which, although not life-threatening, disrupt daily activities through missed work, prolonged antibiotic courses, and increased healthcare utilization. It is recommended that prospective studies of IVIG incorporate quality-of-life instruments to capture patient-reported quality-of-life outcomes.

We also acknowledge that cost-effectiveness is an important consideration with IVIG. Earlier studies raised concerns about the high treatment costs relative to clinical benefit (10). However, the benefit to quality of life may not be as quantifiable as medical costs. Furthermore, there may be subgroups of patients who would most benefit from IVIG and for whom IVIG would be cost-effective and essential.

## Data Availability

The data generated in this study are not publicly available due to patient privacy considerations but are available from the corresponding author on reasonable request.

## References

[1] da Cunha-Bang C , Simonsen J, Rostgaard K, Geisler CH, Hjalgrim H, Niemann C. Improved survival for patients with CLL in the era of combination chemoimmunotherapy - a Danish population based study. Blood 2015;126:1740. 10.1038/bcj.2016.105 PMC5148052 27834937

[2] Andersen MA , Eriksen CT, Brieghel C, Biccler JL, da Cunha-Bang C, Helleberg M, . Incidence and predictors of infection among patients prior to treatment of chronic lymphocytic leukemia: a Danish nationwide cohort study. Haematologica 2018;103:e300–3. 29519862 10.3324/haematol.2017.182006 PMC6029543

[3] Noto A , Cassin R, Mattiello V, Bortolotti M, Reda G, Barcellini W. Should treatment of hypogammaglobulinemia with immunoglobulin replacement therapy (IGRT) become standard of care in patients with chronic lymphocytic leukemia? Front Immunol 2023;14:1062376. 37122737 10.3389/fimmu.2023.1062376 PMC10140292

[4] Otani IM , Lehman HK, Jongco AM, Tsao LR, Azar AE, Tarrant TK, . Practical guidance for the diagnosis and management of secondary hypogammaglobulinemia: a work group report of the AAAAI primary immunodeficiency and altered immune response committees. J Allergy Clin Immunol 2022;149:1525–60. 35176351 10.1016/j.jaci.2022.01.025

[5] Cooperative Group for the Study of Immunoglobulin in Chronic Lymphocytic Leukemia; Gale RP, Chapel HM, Bunch C, Rai KR, Foon K, . Intravenous immunoglobulin for the prevention of infection in chronic lymphocytic leukemia. A randomized, controlled clinical trial. N Engl J Med 1988;319:902–7. 2901668 10.1056/NEJM198810063191403

[6] Boughton BJ , Jackson N, Lim S, Smith N. Randomized trial of intravenous immunoglobulin prophylaxis for patients with chronic lymphocytic leukaemia and secondary hypogammaglobulinaemia. Clin Lab Haematol 1995;17:75–80. 7621634 10.1111/j.1365-2257.1995.tb00322.x

[7] Raanani P , Gafter-Gvili A, Paul M, Ben-Bassat I, Leibovici L, Shpilberg O. Immunoglobulin prophylaxis in hematological malignancies and hematopoietic stem cell transplantation. Cochrane Database Syst Rev 2008;2008:CD006501. 18843719 10.1002/14651858.CD006501.pub2 PMC10936547

[8] Sun C , Tian X, Lee YS, Gunti S, Lipsky A, Herman SEM, . Partial reconstitution of humoral immunity and fewer infections in patients with chronic lymphocytic leukemia treated with ibrutinib. Blood 2015;126:2213–19. 26337493 10.1182/blood-2015-04-639203 PMC4635117

[9] Carrillo de Albornoz S , Zhang X, Arnolda R, MacPhail A, Irving A, Higgins AM, . Immunoglobulin use, survival, and infection outcomes in patients with chronic lymphocytic leukemia. Blood Adv 2025;9:5367–77. 40742276 10.1182/bloodadvances.2025015867 PMC12597636

[10] Weeks JC , Tierney MR, Weinstein MC. Cost effectiveness of prophylactic intravenous immune globulin in chronic lymphocytic leukemia. N Engl J Med 1991;325:81–6. 1904989 10.1056/NEJM199107113250202

